# Increased Risk of Developing Systemic Lupus Erythematosus and Rheumatoid Arthritis in Patients with Primary Sjögren’s Syndrome—A Secondary Cohort Analysis of Nationwide, Population-Based Claims Data

**DOI:** 10.3390/jcm12124157

**Published:** 2023-06-20

**Authors:** Malcolm Koo, Chia-Wen Hsu, Ming-Chi Lu

**Affiliations:** 1 Graduate Institute of Long-Term Care, Tzu Chi University of Science and Technology, Hualien 970302, Taiwan; m.koo@utoronto.ca; 2 Dalla Lana School of Public Health, University of Toronto, Toronto, ON M5T 3M7, Canada; 3 Department of Medical Research, Dalin Tzu Chi Hospital, Buddhist Tzu Chi Medical Foundation, Chiayi 622401, Taiwan; dl50299@tzuchi.com.tw; 4 Division of Allergy, Immunology and Rheumatology, Dalin Tzu Chi Hospital, Buddhist Tzu Chi Medical Foundation, Chiayi 622401, Taiwan; 5 School of Medicine, Tzu Chi University, Hualien 970374, Taiwan

**Keywords:** primary Sjögren’s syndrome, systemic lupus erythematosus, rheumatoid arthritis, National Health Insurance Research Database, rheumatologist

## Abstract

Background: This retrospective cohort study aimed to examine the risk of developing systemic lupus erythematosus (SLE) and rheumatoid arthritis (RA) in patients with primary Sjögren’s syndrome (pSS) compared to controls using data from a nationwide health claims database. Methods: Four distinct cohorts of patients with newly diagnosed pSS were established using Taiwan’s National Health Insurance Research Database. Cohorts I and II were created to evaluate the risk of developing SLE and RA, respectively. Cohorts III and IV were assembled similarly to Cohorts I and II but employed a stricter definition, based on catastrophic illness certificate (CIC) status, for identifying patients with pSS. Comparison cohorts of patients without pSS were formed by frequency matching for sex, 5-year age interval, and index year. Incident rate ratios (IRR) for SLE or RA development were determined using Poisson regression models. Results: Patients with pSS, selected from just outpatient visits or with additional CIC status showed a significantly higher risk of developing SLE or RA compared with the controls. When stratified by age group or sex, the risk of developing SLE was notably higher in the young age group (adjusted IRR 47.24, *p* = 0.002) and women (adjusted IRR 7.63, *p* = 0.003) among patients with pSS. In addition, both men and women with pSS, irrespective of age, showed a significantly elevated risk of developing RA. Conclusions: Patients with pSS exhibited an elevated risk of developing SLE and RA. Rheumatologists should carefully monitor patients with pSS for potential SLE and RA development.

## 1. Introduction

Primary Sjögren’s syndrome (pSS) is a chronic autoimmune disease that predominantly affects women. Its primary clinical symptoms are dry eyes and a dry mouth, and the characteristic pathology involves lymphocytic infiltrates in the affected gland [1]. Sjögren’s syndrome has an estimated prevalence of 0.3 to 1 per 1000 persons [2], with a prevalence of 0.5 per 1000 persons in Taiwan [3]. In addition, patients with pSS may also exhibit systemic manifestations, such as fever, skin vasculitis, lymphadenopathy, or arthritis, and these patients may be associated with other systemic diseases, such as systemic lupus erythematosus (SLE) and rheumatoid arthritis (RA) [4]. Shared genetic backgrounds and environmental or hormonal factors may contribute to the clustering of autoimmune diseases, such as pSS, SLE, and RA [5].

SLE is a systemic autoimmune disease mainly affecting women of childbearing age, with a female-to-male ratio of 9:1. It can attack various organs and systems, leading to severe complications [6]. As a result, patients with SLE have 2- to 3-times higher mortality than controls [6]. RA is also a chronic systemic autoimmune disease characterized by persistent inflammation of the joints, leading to disability [7], which ultimately results in increased mortality in patients with RA [8].

Patients with SLE and pSS share clinical and serological features [9], as well as common genetic background and epigenetic changes [10,11]. Our previous study showed that pSS is often diagnosed before SLE [12] and that 1.3% (6/445) of patients with pSS developed SLE within six years of follow-up [13]. In addition, a meta-analysis showed that patients with pSS might have a higher risk of developing RA [14]. In a retrospective cohort study of 681 patients with pSS, 1% (*n* = 5) developed SLE and 2.1% (*n* = 14) developed RA after 4.7 years of follow-up [15]. On the other hand, one study suggested that pSS patients with articular manifestations might just resemble RA, but it differed in the absence of structural damage [16]. Another study found that none of the 114 patients with pSS developed SLE or RA with ten years of follow-up [17].

The aim of this study is to investigate whether patients with pSS are associated with an elevated risk of developing SLE or RA compared to controls and explore the incidence rates and incidence rate ratios of SLE or RA in patients with pSS compared to controls.

## 2. Materials and Methods

### 2.1. Identification of pSS Cohort I and Comparison Cohort I for Evaluating the Risk of SLE

This retrospective cohort study used the claims data from Taiwan’s National Health Insurance Research Database (NHIRD). It was approved by the institutional review board of the Dalin Tzu Chi Hospital, Buddhist Tzu Chi Medical Foundation, Taiwan (No. B10104020). Patients with pSS were identified from the 2000 Longitudinal Health Insurance Database (LHID 2000), which includes outpatient data files from 1996 to 2000 for one million randomly sampled beneficiaries from all health insurance enrollees in the Registry of Beneficiaries of the NHIRD in 2000. The study included patients aged 20 to 80 years on the index date.

A cohort of patients diagnosed with pSS was assembled based on the International Classification of Diseases, ninth revision, clinical modification (ICD-9-CM) code 710.2 at least three times in the 90 days. The index date for pSS in these patients was defined as the date of their first diagnosis. The European Study Group on Classification Criteria for Sjögren’s Syndrome was used to diagnose pSS [18,19]. The exclusion criteria were patients who had been diagnosed with pSS before 2000 or had been diagnosed with SLE (ICD-9-CM code 710.0) before their pSS diagnosis. This cohort is referred to as pSS Cohort I.

A comparison cohort of individuals without pSS (Comparison Cohort I) was assembled from the LHID 2000. Ten individuals were identified for each patient with pSS based on frequency matching for 5-year age intervals, sex, and index year. Patients who had been diagnosed with SLE prior to the index date were excluded from the study.

Those who had been diagnosed as having systemic autoimmune diseases in the pSS Cohort I and Comparison Cohort I were excluded from the study. The systemic autoimmune diseases examined included thromboangiitis obliterans (ICD-9-CM code 443.1), polyarteritis nodosa (ICD-9-CM code 446.0), hypersensitivity vasculitis (ICD-9-CM code 446.2), Wegener’s granulomatosis (ICD-9-CM code 446.4), giant cell arteritis (ICD-9-CM code 446.5), arteritis obliterans (ICD-9-CM code 446.7), systemic sclerosis (ICD-9-CM code 710.1), polymyositis (ICD-9-CM code 710.4), and dermatomyositis (ICD-9-CM code 710.5).

### 2.2. Identification of SLE

The diagnosis of SLE was confirmed using the 2000−2012 Catastrophic Illness Certificate (CIC) data file. New and successful applicants for the CIC with SLE were defined as patients with newly diagnosed SLE. Individuals diagnosed with SLE prior to or within three months of the index date were excluded from the study. SLE diagnosis was confirmed based on meeting the 1997 American College of Rheumatology (ACR) revised criteria [20].

### 2.3. Identification of Potential Confounding Factors and Comorbidities of SLE

The potential confounding effects of several diseases were examined using respective ICD-9-CM codes within one year prior to the index date. The factors were thyroid diseases, including simple and unspecified goiter (ICD-9-CM 240), thyrotoxicosis (ICD-9-CM 242), hypothyroidism, unspecified (ICD-9-CM 244.9), and thyroiditis (ICD-9-CM 245) [21]. In addition, the comorbidities were identified using respective ICD-9-CM codes as used in our previous study [22].

### 2.4. Identification of pSS Cohort II and Comparison Cohort II for Evaluating the Risk of RA

The methods for identification of patients with pSS for pSS Cohort II were similar to that of pSS Cohort I with some modifications. In addition to a previous diagnosis of pSS, patients diagnosed with RA (ICD-9-CM code 714.0) before diagnosis of pSS were also excluded.

A cohort of individuals without pSS (Comparison Cohort II) was assembled from the LHID 2000, and those diagnosed with RA prior to the index date were excluded. For each pSS patient, ten individuals were identified based on frequency matching for 5-year age intervals, sex, and index year.

### 2.5. Identification of RA

The diagnosis of RA was confirmed using the 2000−2012 CIC data file. New and successful applicants of the CIC with SLE (International Classification of Diseases, ninth revision, clinical modification, ICD-9-CM code 714.0) were defined as patients with newly diagnosed RA. Individuals diagnosed with RA prior to or within three months of the index date were excluded from the study. RA diagnosis was confirmed based on meeting the 1987 American College of Rheumatology revised criteria [23].

### 2.6. Identification of Potential Confounding Factors and Comorbidities of RA

To identify potential confounding factors associated with RA, the presence of thyroid disease as described above and juvenile rheumatoid arthritis (ICD-9-CM codes 714.30, 714.31, 714.32, and 714.33) were examined. Comorbidities associated with RA were also identified using the same method described above.

### 2.7. Identification of pSS Cohorts III and IV and Comparison Cohorts III and IV for Evaluating the Risk of SLE or RA

pSS Cohorts III and IV were formed similarly to pSS Cohorts I and II but employed a stricter definition for identifying pSS patients. pSS Cohort III was assembled with patients identified as having pSS using the 2000−2012 CIC data file. The date of the application of CIC was defined as the index date. We included patients aged 20 to 80 years on the index date and excluded those with a previous diagnosis of SLE. Comparison Cohort III was assembled with patients matched to the pSS patients in a 9:1 ratio, based on 5-year age intervals, sex, and index year from Comparison Cohort I. A total of 3289 patients were selected.

pSS Cohort IV excluded patients with a previous diagnosis of RA and was otherwise similar to pSS Cohort III. Comparison Cohort IV consisted of patients matched to the pSS patients in a 9:1 ratio, based on 5-year age intervals, sex, and index year from Comparison Cohort II. A total of 3233 patients were selected.

### 2.8. Statistical Analysis

The clinical parameters of the patients in the pSS and comparison cohorts were compared using a t-test or Chi-square test. Incidence rate ratios (IRRs) for the outcome variables were calculated using Poisson regression models (generalized linear models with a Poisson log-linear link function and person–years as the offset variable), with and without adjusting for potential confounding factors. Subgroup analyses were performed with stratification by age group and gender. All statistical analyses were performed using the IBM SPSS Statistics software package, version 24.0 (IBM Corp, Armonk, NY, USA). A *p*-value of <0.05 was considered significant.

## 3. Results

### 3.1. Risk of SLE in Patients with pSS

Table 1 shows the basic characteristics of 2096 patients with pSS (pSS Cohort I) and 21,213 controls (Comparison Cohort I). The mean age of patients with pSS was 54.7 (standard deviation 14.8 years), and 79.2% were women. In addition, 65.7% of the patients were in the 50–80 years age interval. Patients with pSS had a significantly higher socioeconomic condition, as defined by insurance premiums based on salary, compared to the controls. The geographical region was significantly different between the pSS patients and the comparison cohort. As expected, patients with pSS had a higher prevalence of thyroid disease (1.5% vs. 0.4%; *p* < 0.001), and musculoskeletal disorders (21.8% vs. 1.9%; *p* = 0.001). Patients with pSS also had a significantly higher prevalence of several other conditions, including hypertension, chronic obstructive pulmonary disease (COPD), malignancy, hyperlipidemia, coronary artery disease (CAD), peripheral vascular disease (PVD), cerebrovascular accident (CVA), peptic ulcer disease (PUD), and liver disease.

Table 2 shows the incidence rates and incidence rate ratios (IRRs) for developing SLE in pSS Cohort I and Comparison Cohort I, with and without stratification by sex and age group. Overall, patients with pSS had a significantly higher risk of developing SLE compared to Comparison Cohort I (adjusted IRR 10.10; 95% CI: 2.78–36.75, *p* < 0.001). Only women with pSS had a significantly elevated incidence of developing SLE (adjusted IRR 7.63; 95% CI: 1.97–29.56, *p* = 0.003). Younger patients (age 20–49 years) with pSS showed an exceptionally high incidence ratio for developing SLE (adjusted IRR 47.24; 95% CI: 4.27–522.18, *p* = 0.002), while the risk of developing SLE was not significantly elevated in older patients with pSS (age 50–80 years).

### 3.2. Risk of RA in Patients with pSS

Table 3 shows the basic characteristics of 2083 patients with pSS (pSS Cohort II) and 20,830 controls (Comparison Cohort II). Patients with pSS had a significantly higher socioeconomic status, as estimated by insurance premiums based on salary, compared to the controls. The geographical region was significantly different between the pSS patients and the comparison cohort. Patients with pSS had a higher prevalence of thyroid disease (1.6% vs. 0.4%; *p* < 0.001) and musculoskeletal disorders (21.2% vs. 1.9%; *p* < 0.001). In addition, patients with pSS showed a significantly higher prevalence of several other conditions, including hypertension, COPD, malignancy, hyperlipidemia, CAD, PVD, CVA, PUD, chronic kidney disease, and liver disease.

Table 4 presents the incidence rates and incidence rate ratios (IRRs) for developing RA in patients with pSS and Comparison Cohort II. Patients with pSS had a significantly higher risk of developing RA compared with Comparison Cohort II (adjusted IRR 17.13; 95% CI: 8.82–33.24, *p* < 0.001). Both men (adjusted IRR 28.61; 95% CI: 2.46–332.58, *p* = 0.007) and women (adjusted IRR 17.81; 95% CI: 8.90–35.62, *p* < 0.001) with pSS had an elevated incidence of developing RA. Both older (age 50–80 years; adjusted IRR 19.96; 95% CI: 9.11–43.70, *p* < 0.001) and younger patients (age 20–49 years; adjusted IRR 12.01; 95% CI: 3.38–42.63, *p* < 0.001) with pSS had a higher risk of developing RA.

### 3.3. Risk of SLE and RA in Patients with pSS Based on the CIC Database

Because the NHIRD included the diagnosis of pSS in the CIC data file, we performed an additional analysis with a stricter definition for identifying patients with pSS, based on their CIC status. Respective Comparison Cohorts III and IV were generated from Comparison Cohort I and II, respectively. Table 5 shows that patients with pSS had a significantly higher risk of developing SLE (adjusted IRR 11.74; 95% CI: 2.79–49.32, *p* = 0.001) and RA (adjusted IRR 19.34; 95% CI: 7.49–49.91, *p* < 0.001). However, the number of newly diagnosed SLE and RA cases in the pSS Cohorts III and IV was too low to perform further subgroup analysis stratified by age or sex.

## 4. Discussion

In this retrospective cohort study, we demonstrated that young women with pSS were at an exceptionally high risk of developing SLE. In contrast, both men and women with pSS, regardless of age, had a significantly higher risk of developing RA. These findings are consistent with previous studies. Those who had arthralgias and arthritis and positive serology for rheumatoid factor or anti-cyclic citrullinated peptide antibodies were more likely to develop RA [15]. Kuo et al. showed that the risk of pSS and other autoimmune diseases, such as RA or SLE, was higher in the relatives of patients with pSS [3]. Common genetic characteristics, particularly genes involved in the regulation of T cell activation and the T cell receptor (TCR) signaling pathway, have been linked to familial aggregation of pSS, SLE, and RA [24]. In addition, the activation of interferon (IFN) type I pathways is commonly observed in patients with pSS, SLE, and RA, and the genetic variants of its downstream molecules, such as STAT4, are associated with the risk of these diseases [10]. Given that the onset of SLE is generally among women of childbearing age and up to 90% of patients with SLE are female, it is not surprised that women with pSS in the age group of 20 to 49 had a higher risk of developing SLE. Among the pSS patients, those who had Raynaud’s phenomenon, arthritis, and kidney involvement were likely to be diagnosed with SLE [9].

It should be noted that over two thousand patients with pSS were identified using the selection criteria of ICD-9-CM code 710.2 three times during the 90 days. However, when a stricter selection criterion based on the CIC data file was used, only 710 patients with pSS could be identified. We propose several explanations for this discrepancy. First, for patients to successfully obtain a CIC for pSS, they must meet the American–European classification criteria for Sjögren’s syndrome. For those without detectable serum autoantibodies to Ro (SSA) or La (SSB) antigen, a pathology report of focal lymphocytic sialoadenitis must be obtained and evaluated by an expert histopathologist, with a focus score of >1. This involves an invasive minor salivary gland biopsy procedure that many patients refuse to undergo [25]. In addition, this procedure has variable sensitivity, specificity, and low reproducibility [26]. Second, according to a study performed using the NHIRD, the mortality rate for patients with pSS was not significantly higher than that of the general population of Taiwan [27]. Therefore, rheumatologists may be less likely to apply for a CIC. Despite these limitations, we performed an additional analysis in this study using the patients with pSS with CIC status. We found that patients with pSS had an elevated risk of developing SLE or RA, but the case number was too small for further subgroup analysis. In contrast to patients with pSS, patients with SLE have a markedly higher mortality rate, and patients with RA have a high mortality rate and a high probability of disability. Therefore, it is crucial to identify these diseases as early as possible and initiate appropriate treatment.

We noted that there are some limitations to this study. First, due to the limitation of NHIRD, serological data, clinical parameters, and disease activity for patients with pSS could not be accessed. Second, pSS, SLE, and RA identification were based solely on ICD-9-CM codes, which might have led to misclassification. Nevertheless, the National Health Insurance Administration conducts regular audits to ensure the accuracy of medical claims. The database we used in this study ranges from January 1999 to December 2012. Most of the patients with pSS, SLE, and RA were diagnosed according to the older version of the classification criteria. Finally, patients with pSS might receive immunosuppressive therapy which might affect the occurrence rate of SLE or RA. More studies are needed to clarify these issues.

## 5. Conclusions

Our findings highlight the importance of monitoring patients with pSS, especially young women, for the potential development of SLE. Moreover, all patients with pSS should be closely monitored for the increased risk of developing RA. Given the significant morbidity and mortality associated with SLE and RA, close monitoring and early intervention, such as pharmacological treatment and lifestyle modifications, are imperative for the pSS population.

## Figures and Tables

**Table 1:** Basic characteristics of primary Sjögren’s syndrome Cohort I and Comparison Cohort I (*n* = 23,309).

Variable	*n* (%)				*p*-Value
	Primary Sjögren’s Syndrome Cohort I 2096 (9.0)		Comparison Cohort I 21,213 (91.0)		
Sex					0.975
Male	437	(20.8)	4429	(20.9)	
Female	1659	(79.2)	16,784	(79.1)	
Age interval at entry (years)					0.923
20–49	719	(34.3)	7299	(34.4)	
50–80	1377	(65.7)	13,914	(65.6)	
Mean age (standard deviation) (years)	54.7	(14.8)	54.7	(14.8)	0.957
Median (quartile 1–3) (years)	55	(45–67)	55	(45–67)	
Socioeconomic status (*n* = 23,287)					<0.001
Low	1042	(49.7)	11,696	(55.2)	
Middle	672	(32.1)	6497	(30.7)	
High	382	(18.2)	2998	(14.1)	
Geographical region (*n* = 22,662)					<0.001
Northern	1034	(50.8)	12,599	(61.1)	
Central	637	(31.3)	3140	(15.2)	
Southern	345	(16.)	4444	(21.5)	
Eastern	20	(1.0)	443	(2.2)	
Thyroid diseases	32	(1.5)	83	(0.4)	<0.001
Comorbidities					
Hypertension	591	(28.2)	4449	(21.0)	<0.001
Diabetes mellitus	235	(11.2)	2189	(10.3)	0.202
Congestive heart failure	43	(2.1)	377	(1.8)	0.368
Chronic obstructive pulmonary disease	335	(16.0)	1791	(8.4)	<0.001
Malignancy	112	(5.3)	800	(3.8)	0.001
Hyperlipidemia	382	(18.2)	2138	(10.1)	<0.001
Coronary artery disease	50	(2.4)	220	(1.0)	<0.001
Prior myocardial infarction	7	(0.3)	60	(0.3)	0.677
Peripheral vascular disease	25	(1.2)	136	(0.6)	0.004
Cerebrovascular accident	147	(7.0)	969	(4.6)	<0.001
Dementia	16	(0.8)	166	(0.8)	0.924
Musculoskeletal disorders	456	(21.8)	395	(1.9)	0.001
Peptic ulcer disease	440	(21.0)	2025	(9.5)	<0.001
Chronic kidney disease	73	(3.5)	597	(2.8)	0.081
Liver disease	232	(11.1)	1127	(5.3)	<0.001

Socioeconomic status was estimated by insurance premiums based on salary. Low: <19,000 New Taiwan dollars (TWD); middle: 19,001–24,000; and high: >24,000.

**Table 2:** The incidence rate and incidence risk ratio of systemic lupus erythematosus in primary Sjögren’s syndrome Cohort I and Comparison Cohort I.

Stratification	Primary Sjögren’s Syndrome Cohort I (*n* = 2096)		Comparison Cohort I (*n* = 21,213)		IRR (95% CI) *p*-Value	aIRR * (95% CI) *p*-Value
	No. of Patients	IR	No. of Patients	IR		
Overall	10	9.36	5	0.48	19.49 (6.66–57.02) <0.001	10.10 (2.78–36.75) <0.001
Sex						
Male	1	4.39	0	n.c.	n.c.	n.c.
Female	9	10.70	5	0.61	17.65 (5.92–52.66) <0.001	7.63 (1.97–29.56) 0.003
Age group (years)						
20–49	7	17.62	1	0.24	72.49 (8.92–589.18) <0.001	47.24 (4.27–522.18) 0.002
50–80	3	4.47	4	0.63	7.04 (1.58–31.44) 0.011	3.82 (0.60–24.06) 0.154

aIRR: adjusted incidence rate ratio; CI: confidence interval; n.c.: not calculable; IR: incidence rate per 10,000 person–years; IRR: incidence rate ratio. * Adjusted for age, sex, socioeconomic status, geographic region, thyroid diseases, and all comorbidities.

**Table 3:** Basic characteristics of primary Sjögren’s syndrome Cohort II and Comparison Cohort II (*n* = 22,913).

Variable	*n* (%)				*p*-Value
	Primary Sjögren’s Syndrome Cohort II 2083 (9.1)		Comparison Cohort II 20,830 (90.9)		
Sex					>0.999
Male	435	(20.9)	4350	(20.9)	
Female	1648	(79.1)	16,480	(79.1)	
Age interval at entry (years)					>0.999
20–49	740	(35.5)	7400	(35.5)	
50–80	1343	(64.5)	13,430	(64.5)	
Mean age (standard deviation) (years)	54.29	(15.06)	54.28	(15.06)	0.986
Median (quartile 1–3) (years)	55	(44–67)	55	(44–67)	
Socioeconomic status (*n* = 22,891)					<0.001
Low	1038	(49.8)	11,663	(56.1)	
Middle	662	(31.8)	6212	(29.9)	
High	383	(18.4)	2933	(14.0)	
Geographical region (*n* = 22,307)					<0.001
Northern	1031	(51.0)	12,349	(60.9)	
Central	634	(31.4)	3198	(15.8)	
Southern	338	(16.7)	4279	(21.1)	
Eastern	18	(0.9)	460	(2.2)	
Thyroid diseases	33	(1.6)	77	(0.4)	<0.001
Juvenile rheumatoid arthritis	0	(0.0)	0	(0.0)	
Comorbidities					
Hypertension	590	(28.3)	4313	(20.7)	<0.001
Diabetes mellitus	228	(10.9)	2110	(10.1)	0.241
Congestive heart failure	42	(2.0)	370	(1.8)	0.432
Chronic obstructive pulmonary disease	340	(16.3)	1730	(8.3)	<0.001
Malignancy	113	(5.4)	843	(4.0)	0.003
Hyperlipidemia	379	(18.2)	2177	(10.5)	<0.001
Coronary artery disease	50	(2.4)	202	(1.0)	<0.001
Prior myocardial infarction	7	(0.3)	66	(0.3)	0.838
Peripheral vascular disease	26	(1.2)	165	(0.8)	0.029
Cerebrovascular accident	146	(7.0)	960	(4.6)	<0.001
Dementia	17	(0.8)	173	(0.8)	0.945
Musculoskeletal disorders	441	(21.2)	402	(1.9)	<0.001
Peptic ulcer disease	419	(20.1)	1932	(9.3)	<0.001
Chronic kidney disease	84	(4.0)	619	(3.0)	0.007
Liver disease	230	(11.0)	1070	(5.1)	<0.001

Socioeconomic status was estimated by insurance premiums based on salary. Low: <19,000 New Taiwan dollars (TWD); middle: 19,001–24,000; and high: >24,000.

**Table 4:** The incidence rate and incidence risk ratio of rheumatoid arthritis in primary Sjögren’s syndrome Cohort II and Comparison Cohort II.

Stratification	Primary Sjögren’s Syndrome Cohort II (*n* = 2083)		Comparison Cohort II (*n* = 20,830)		IRR (95% CI) *p*-Value	aIRR * (95% CI) *p*-Value
	No. of Patients	IR	No. of Patients	IR		
Overall	46	44.74	15	1.48	30.30 (16.92–54.27) <0.001	17.13 (8.82–33.24) <0.001
Sex						
Male	7	31.83	1	0.47	67.38 (8.29–547.67) <0.001	28.61 (2.46–332.58) 0.007
Female	39	48.25	14	1.73	27.72 (15.05–51.04) <0.001	17.81 (8.90–35.62) <0.001
Age group (years)						
20–49	16	39.33	5	1.20	32.77 (12.00–89.45) <0.001	12.01 (3.38–42.63) <0.001
50–80	30	48.28	10	1.67	28.94 (14.14–59.19) <0.001	19.96 (9.11–43.70) <0.001

aIRR: adjusted incidence rate ratio; CI: confidence interval; IR: incidence rate per 10,000 person–years; IRR: incidence rate ratio. * Adjusted for age, sex, socioeconomic status, geographical region, thyroid diseases, juvenile rheumatoid arthritis, and all comorbidities.

**Table 5:** The incidence rate and incidence risk ratio of systemic lupus erythematosus in primary Sjögren’s syndrome Cohort III and Comparison Cohort III and the incidence rate and incidence risk ratio of rheumatoid arthritis in primary Sjögren’s syndrome Cohort IV and Comparison Cohort IV.

No. of Patients	IR	No. of Patients	IR	IRR (95% CI) *p*-Value	aIRR * (95% CI) *p*-Value
Systemic lupus erythematosus (710.0)					
Primary Sjögren’s syndrome Cohort III *n* = 710		Comparison Cohort III *n* = 5980			
5	15.20	3	1.18	12.85 (3.07–53.80) <0.001	11.74 (2.79–49.32) 0.001
Rheumatoid arthritis (714.0)					
Primary Sjögren’s syndrome Cohort IV *n* = 710		Comparison Cohort IV *n* = 5940			
15	46.40	6	2.37	19.62 (7.61–50.55) <0.001	19.34 (7.49–49.91) < 0.001

aIRR: adjusted incidence rate ratio; CI: confidence interval; IR: incidence rate per 10,000 person-years; IRR: incidence rate ratio. * Adjusted for age and sex.

## Data Availability

The data are not publicly available due to the Taiwan Personal Information Protection Act. The data that support the findings of this study are available from the corresponding author upon request.
